# Global hotspot and trend of extracorporeal membrane oxygenation for pulmonary embolism

**DOI:** 10.3389/fmed.2025.1531716

**Published:** 2025-03-18

**Authors:** Wei Wang, Jianyu Ji, Lin Han, Jing Pang, Li Mo, Fang Liu, Yamin Gao, Bin Xiong, Shulin Xiang

**Affiliations:** ^1^Guangxi Academy of Medical Sciences, Nanning, Guangxi, China; ^2^Department of Intensive Care Unit, The Peoples Hospital of Guangxi Zhuang Autonomous Region, Nanning, Guangxi, China; ^3^Research Center of Communicable and Severe Diseases, Guangxi Academy of Medical Sciences, Nanning, Guangxi, China; ^4^Guangxi Health Commission Key Laboratory of Diagnosis and Treatment of Acute Respiratory Distress Syndrome, Nanning, Guangxi, China

**Keywords:** extracorporeal membrane oxygenation, pulmonary embolism, CiteSpace, VOSviewer, bibliometrics

## Abstract

**Background:**

Research on extracorporeal membrane oxygenation (ECMO)-assisted support for pulmonary embolism (PE) has been increasing, yet no systematic bibliometric analysis has been conducted. This study evaluates global research trends in this field by analyzing countries, institutions, authors, journals, references, and keywords.

**Methods:**

Relevant articles and reviews published up to August 15, 2023, were retrieved from the Web of Science Core Collection (WOSCC). VOSviewer and CiteSpace software were used for bibliometric analysis of collected data.

**Results:**

Publications on ECMO-assisted support for PE surged from 2015 to 2023, comprising 82.7% (306/370) of total studies. The United States, Germany, and China contributed 62.97% (233/370) of the research. *Perfusion-UK* had the most publications, while *Journal of the American College of Cardiology* was the most cited journal. The University of Maryland, Massachusetts General Hospital, and Harvard Medical School were the leading institutions. Chetan Pasrija published the highest number of papers, while Konstantinidis SV was the most co-cited author. Research hot spots include: (1) ECMO management and survival rates, (2) combined treatments with thrombolysis or surgical thrombectomy, (3) anticoagulation and clot formation, and (4) ECMO support in COVID-19.

**Conclusion:**

This study aims to increase awareness of research hot spots on ECMO-assisted support for PE by determining the collaboration and impact of authors, countries, institutions, and journals. In addition, it comprehensively reviews research trends on ECMO regarding PE. It also provides a reference for potential collaborators, institutions, and future research prospects.

## Introduction

Acute pulmonary embolism (PE), a clinical and pathological syndrome, is characterized by sudden obstruction of the pulmonary artery or its branches owing to exogenous or endogenous thrombi, leading to significant disturbances in pulmonary circulation (1). Epidemiologically, PE presents an incidence rate of 60–120 cases per 100,000 individuals annually worldwide (2). In the United States, PE accounts for an estimated 60,000–100,000 fatalities each year (3). Moreover, emerging evidence suggests an increased risk of PE associated with the COVID-19 virus (4).

Contemporary clinical guidelines recommend systemic thrombolysis or surgical thrombectomy as reperfusion therapies in high-risk patients with PE. However, in critically ill patients presenting with shock or heart failure, thrombolysis needs to be conducted for a certain duration and intensity to be effective. Although surgical thrombectomy can be curative for PE, its success rate is notably lower in patients suffering from cardiogenic shock (5, 6). Multiple case series and meta-analyses have underscored the pivotal role of extracorporeal membrane oxygenation (ECMO) in managing PE. Venous–arterial ECMO (V-A ECMO) has been particularly instrumental in providing temporary hemodynamic stabilization, thereby serving as a bridge to surgical or thrombolytic thrombectomy in patients with PE (7, 8).

Bibliometrics is an innovative methodology for assessing the academic impact of scientific publications. It is used to conduct a comprehensive evaluation of the qualitative and quantitative attributes of scholarly literature by examining metrics, such as geographic distribution of research, journal impact, institutional contributions, and thematic keywords. Hence, bibliometrics helps in delineating current trends and identifying emerging frontiers in specialized domains (9). Its efficacy has been substantiated across diverse biomedical disciplines, such as inflammation, immunology, and oncology (10, 11).

Bibliometric analyses have contributed significantly to the development of disease-treatment protocols and clinical guidelines (12). These analyses synthesize research focus, pinpoint nascent trends, and elucidate patterns of collaboration within the corpus of published literature. Moreover, bibliometric methods are instrumental in identifying under-researched topics, thereby furnishing valuable insights and strategic guidance for both researchers and academic institutions.

In recent years, researchers have been paying increasingly more attention to the field of ECMO-assisted support for PE. As a result, numerous studies in this field have been published. Therefore, it becomes critical to summarize the research in this field to gain insights into the current developments. However, no bibliometrics study has yet been conducted to support the publication of articles in the field of ECMO-assisted support for PE. Hence, there is a compelling need to carry out a bibliometric analysis of the existing research on ECMO-assisted interventions for PE, to further elucidate and expand upon this topic.

## Methods

### Data collection

**Figure 1:**
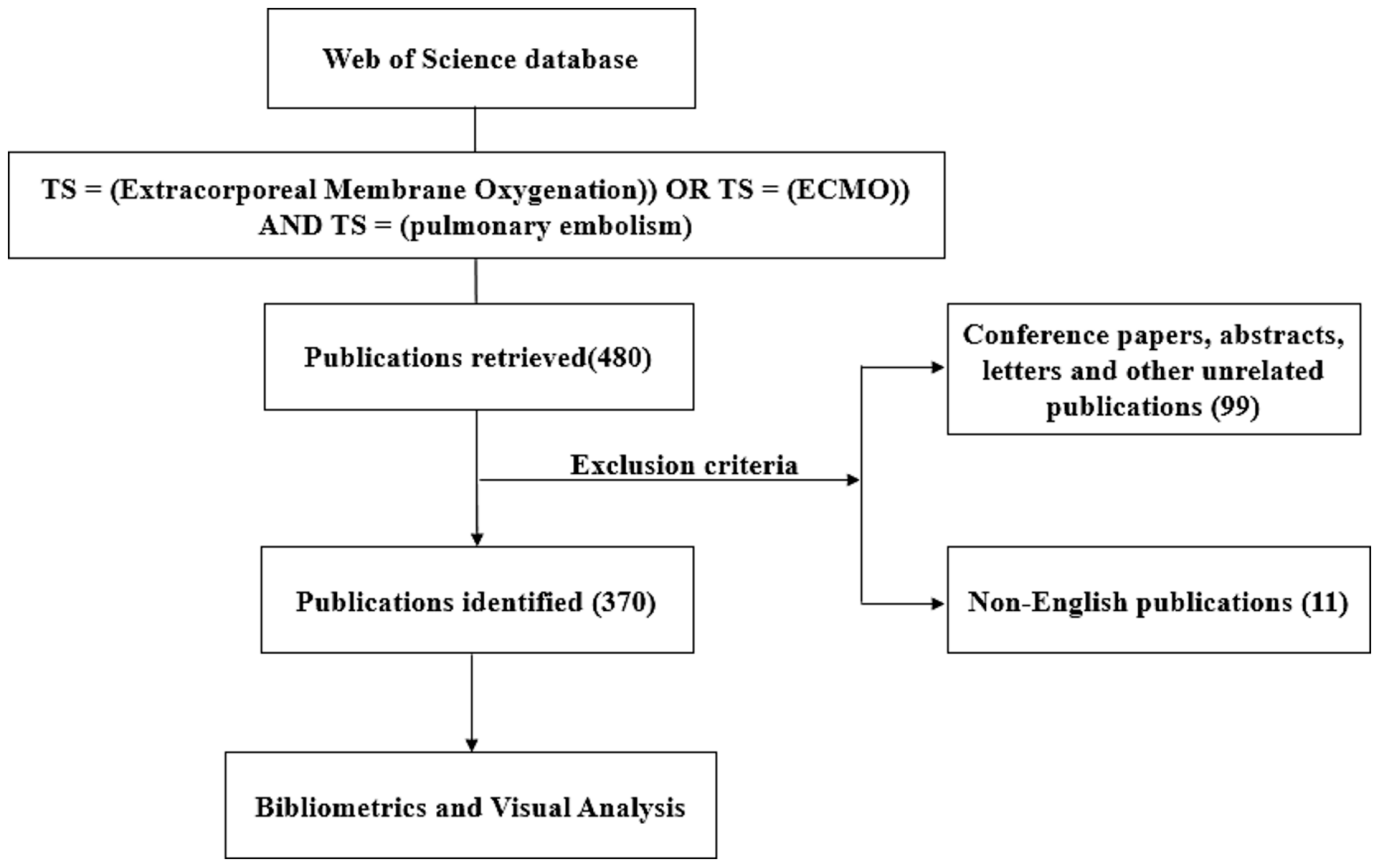
Flow chart of bibliometric analysis of ECMO for PE.

The Web of Science Core Collection (WOSCC) database, a prominent multidisciplinary academic bibliographic resource, was utilized for data retrieval. The following search strategy was employed: #1((TS = (Extracorporeal Membrane Oxygenation)) OR TS = (ECMO)) AND TS = (pulmonary embolism). This approach was specifically designed to capture a broad spectrum of relevant literature. Inclusion Criteria: English-language literature published from 1995 to 2023, including original research articles and review articles. Exclusion Criteria: Non-English literature, conference abstracts, book chapters, and other non-peer-reviewed publications (Figure 1).

The initial screening and selection of references were meticulously conducted by two authors (WW and XSL). Discrepancies encountered during this process were diligently resolved through a consensus-building meeting. The resulting data were systematically downloaded in full-text format, ensuring comprehensive coverage and analysis. Each file was methodically recorded and named in plain text format, adhering to the specific file naming convention “Download*.txt” to facilitate subsequent analysis using CiteSpace 6.2.4, a software program known for its specificity in file name recognition.

### Data processing

VOSviewer, a software program developed by Nees Jan and Ludo (53), facilitates the creation, visualization, and exploration of bibliometric maps based on web data. Utilizing VOSviewer version 1.6.18, we generated knowledge maps encompassing various bibliometric indicators, such as published journals, contributing countries, production journals, co-cited journals, authors, co-cited authors, and related literature. Additionally, we constructed keyword co-occurrence and clustering graphs based on textual data to further elucidate thematic concentrations.

CiteSpace, another major tool for bibliometric and visual analyses, allows the detection of collaborations, key points, internal structures, and evolving trends within a research field (13). We employed CiteSpace version 6.2.4 to focus on visualizing the co-occurrence of countries and institutions, dual journal maps, trends in high-frequency keywords, as well as citations and bursts of references. We configured CiteSpace by considering the period of 1995–2023, with specific parameters for pruning (minimum spanning tree and pruned slice network) and selection criteria (top *N* = 25), while maintaining other settings at their default configurations.

### Data analysis

Microsoft Office Excel 2019 was employed for database management and analysis of annual publication trends. We accessed the 2019 Impact Factor (IF) and Journal Citation Reports (JCR) categories from the Science Network Journal Citation Report published on August 15, 2023. All data utilized in this study were sourced from publicly available databases. Consequently, this study did not need ethical approval.

### Statistical analysis

This study was descriptive and did not involve any statistical analysis methods.

## Results

### Trends in annual publication

**Figure 2:**
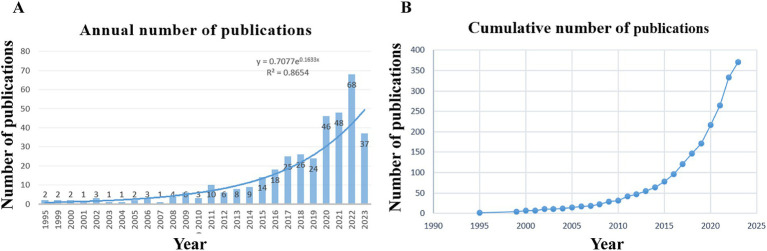
Observed trends in articles published per year. **(A)** Number of publications published each year and projected future trends. **(B)** Cumulative number of publications issued.

The data-retrieval strategy yielded a total of 480 articles. A rigorous screening of these articles eliminated 99 articles, including non-reviews and monographs, from the analysis. Eleven articles not written in English were also omitted. Consequently, the final dataset comprised 370 articles that conformed to the inclusion criteria, spanning the period from 1995 to 2023. Notably, the dataset revealed a marked upward trajectory in the volume of publications related to ECMO-assisted interventions for PE over the last 8 years (2015–2022). The year 2022 alone witnessed the publication volume nearly quintuple to that of 2015, as illustrated in Figure 2. Based on the trend analysis results, we can anticipate a continued increase in the number of publications in this field in the coming years.

### Global collaboration: countries/regions and institutions

**Figure 3:**
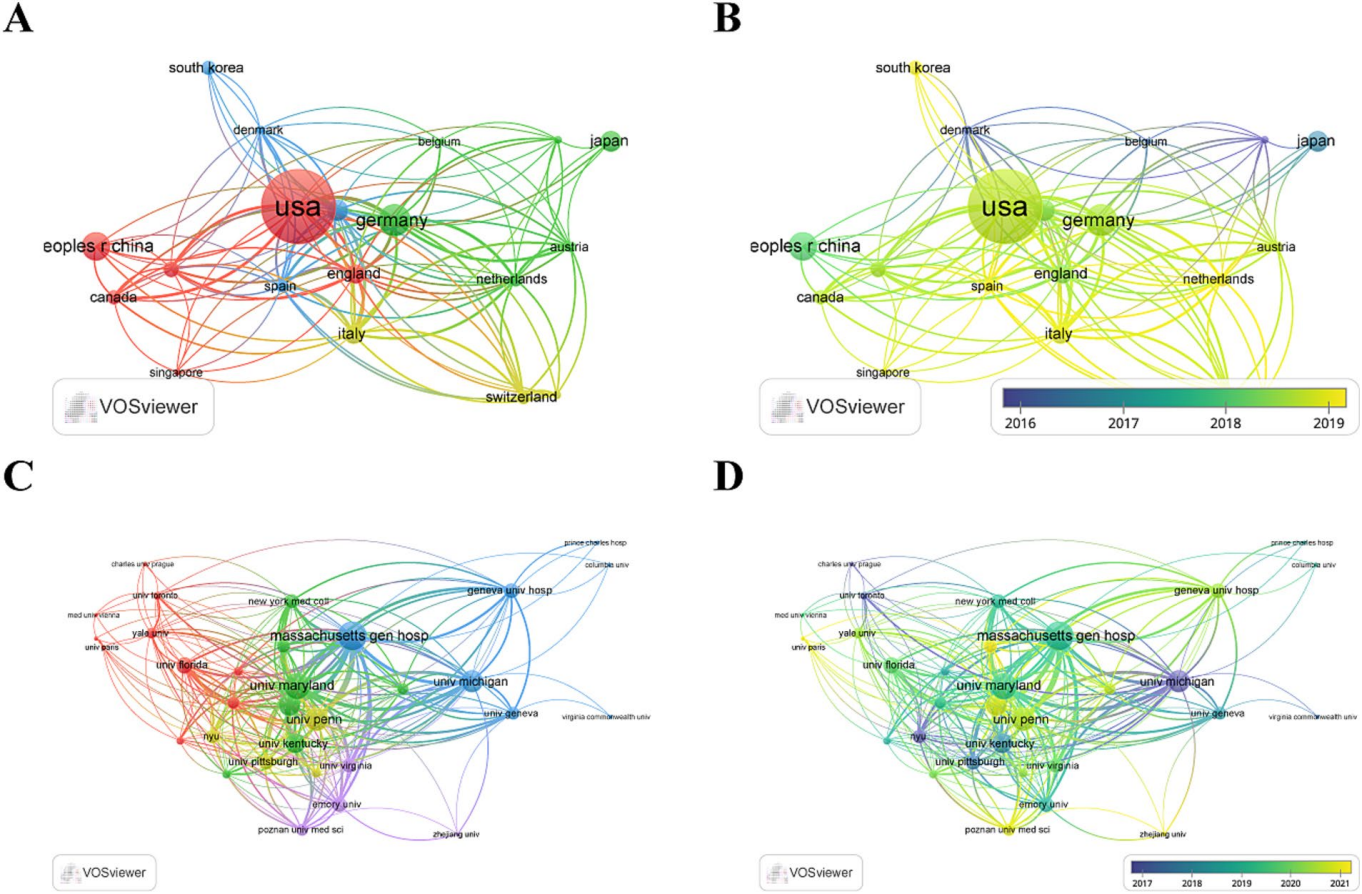
**(A,B)** Visual map of countries generated by VOSviewer (number of articles published 5 ≥ countries); **(C,D)** Visual map of institutions generated by VOSviewer (institutions with a publication threshold of ≥30 articles).

Global collaboration is essential for the further development of this research field. As shown in Figures 3A,B, collaboration among 47 countries/regions and 703 institutions across the world resulted in the publication of 370 papers. The United States led this list with 146 papers, constituting 42.9% of the total, followed by Germany (47 papers, 12.70%), China (40 papers, 10.81%), and France (27 papers, 7.30%) (Table 1). Notably, the United States, which occupies the central position in the global collaboration network, predominantly published its papers after 2019. According to the total international score (TIS), the countries/regions with the maximum collaborations were the United States (84), Germany (73), Italy (60), and the United Kingdom (56), highlighting a dynamic interplay of international cooperation.

**Table 1:** Top 10 prolific countries/regions.

Rank	Country	Documents	TC	AAC	TLS
1	USA	146	4,578	31.4	64
2	Germany	47	5,474	116.5	73
3	China	40	2,198	55.0	12
4	France	27	6,931	256.7	51
5	Japan	26	421	16.2	6
6	Italy	24	5,102	212.6	60
7	England	23	4,963	215.8	56
8	Australia	18	1,934	107.4	26
9	Canada	16	2,467	154.2	24
10	South Korea	16	133	8.3	5

Employing VOSviewer, we constructed an institutional network map by selecting 32 institutions from the abovementioned pool of 703, based on a publication threshold of four or more papers between 1995 and 2023 (Figures 3C,D). The top 10 institutions in terms of publication count included Massachusetts General Hospital (10 papers), Harvard Medical School (9 papers), University of Michigan (9 papers), and University of Pennsylvania (9 papers) (Table 2). Among these leading institutions, the majority are based in the United States (8 out of 10), with the Czech Republic and France each contributing one institution to the top 10.

**Table 2:** Top 10 institutions.

Rank	Organization	Country	Documents	TC	AAC	TLS
1	University of Maryland	USA	12	359	29.9	4
2	Massachusetts General Hospital	USA	10	377	37.7	7
3	Harvard Medical School	USA	9	244	27.1	15
4	Emory University	USA	9	248	27.6	14
5	University of Michigan	USA	9	460	51.1	8
6	University of Pennsylvania	USA	7	328	46.9	15
7	Sorbonne University	France	7	102	14.6	7
8	Charles University in Prague	Czech	7	563	80.4	3
9	University of Pittsburgh	USA	6	361	60.2	14
10	University of Kentucky	USA	6	242	40.3	9

### Analysis of journals and co-cited journals

**Figure 4:**
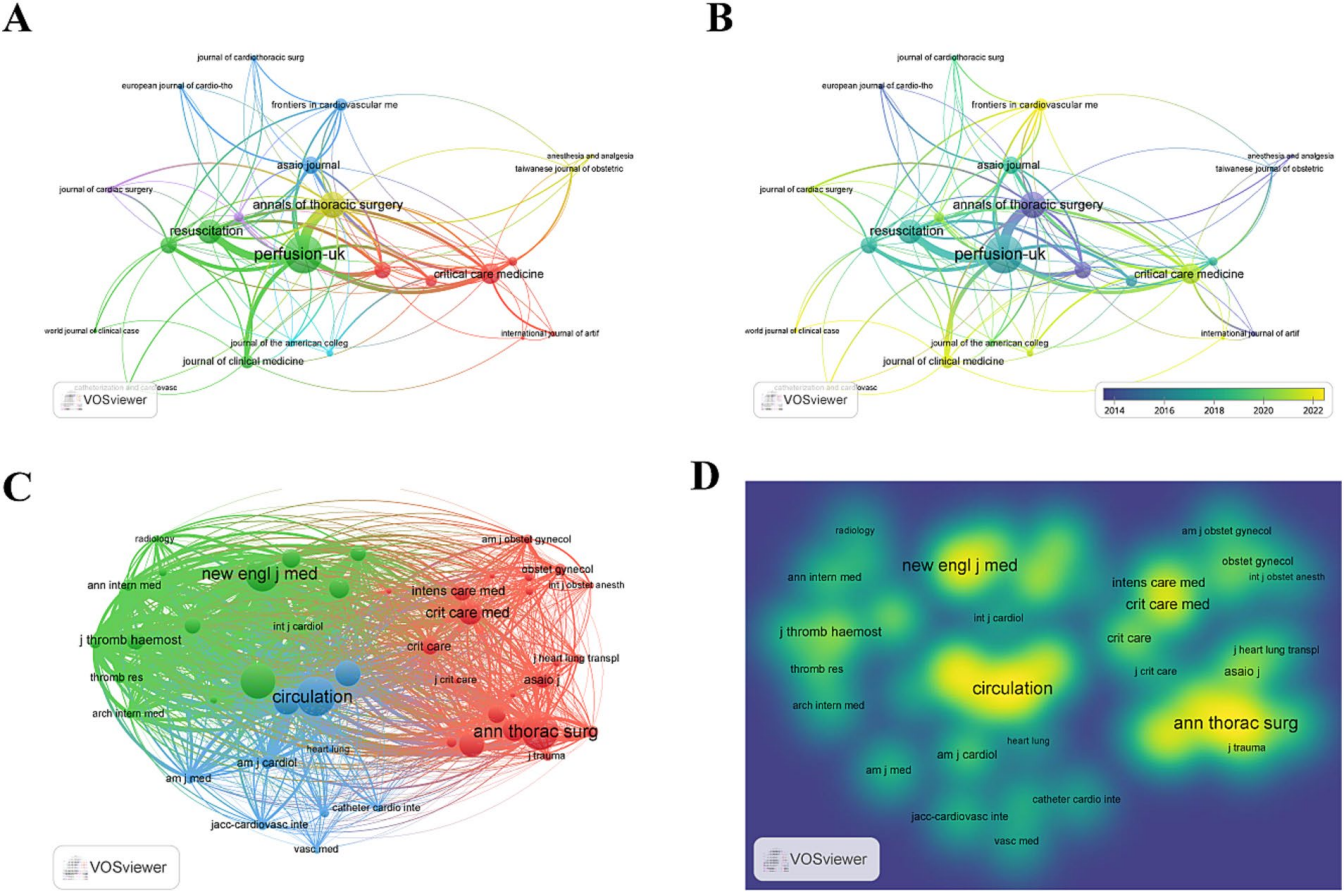
**(A,B)** Visual map of journals generated by VOSviewer (journals with ≥4 publications); **(C,D)** Visual map of co-cited journals generated by VOSviewer (co-citation count ≥50).

We again employed VOSviewer to conduct a co-citation analysis and screen co-cited journals to determine the most prominent and influential publications in this domain (Figures 4A,B). Our findings revealed that 370 articles were published on the topic across 23 academic journals. Leading this publication count was *Perfusion-UK* with 28 articles (3.21%), followed by *ASAIO Journal*, *Journal of Cardiothoracic and Vascular Anesthesia*, *Resuscitation*, and *The Annals of Thoracic Surgery*. In terms of citations, *Perfusion-UK* again topped the list, closely followed by *Critical Care.* Among the top 10 journals, two boasted an IF of >5, while three journals belong to JCR Q1 (Table 3).

**Table 3:** Top 10 journals.

Rank	Journal	Country	IF (2022)	JCR (2022)	Documents	Citations	AAC	TLS
1	Perfusion-UK	ENGLAND	1.200	Q4	28	362	12.9	106
2	Asaio Journal	USA	4.200	Q3	14	273	19.5	35
3	Journal of Cardiothoracic and Vascular Anesthesia	ENGLAND	2.800	Q4	11	50	4.5	17
4	Resuscitation	Netherlands	6.500	Q2	10	249	24.9	57
5	Annals of Thoracic Surgery	Netherlands	4.600	Q2	9	341	37.9	63
6	Critical Care Medicine	USA	8.800	Q1	8	151	18.9	45
7	American Journal of Emergency Medicine	ENGLAND	3.600	Q4	7	53	7.6	21
8	Journal of Cardiothoracic Surgery	ENGLAND	1.600	Q4	7	22	3.1	10
9	World Journal of Clinical Case	USA	1.100	Q4	7	3	0.4	4
10	Seminars in Respiratory and Critical Care Medicine	USA	3.200	Q3	6	71	11.8	15

In a broader scope, among the 1,685 co-cited journals with over 48 citations each, 48 journals exceeded the 50-citation mark. *Circulation* led this group with 596 citations, followed by *The Annals of Thoracic Surgery* (557 citations), *Chest* (523 citations), and *New England Journal of Medicine* (503 citations) (Figures 4C,D; Table 4). Notably, seven of the top 10 most-cited journals were classified within the JCR Q1 category, each with an IF exceeding 8. Five of these leading journals are based in the United States, three in Netherlands, and two in the United Kingdom.

**Table 4:** Top 10 co-cited journals.

Rank	Journal	Country	IF (2022)	JCR (2022)	Citations	TLS
1	Circulation	USA	37.800	Q1	596	35,626
2	Annals of Thoracic Surgery	Netherlands	4.600	Q2	557	17,109
3	Chest	USA	9.600	Q1	523	31,973
4	New England Journal of Medicine	USA	158.5	Q1	503	36,322
5	European Heart Journal	ENGLAND	39.300	Q4	369	23,762
6	Resuscitation	Netherlands	6.500	Q2	352	12,609
7	Journal of The American College of Cardiology	Netherlands	24.000	Q1	344	21,637
8	Critical Care Medicine	USA	8.800	Q1	323	14,063
9	Journal of Thoracic and Cardiovascular Surgery	USA	6.000	Q1	308	12,621
10	Lancet	ENGLAND	168.900	Q1	268	16,401

**Figure 5:**
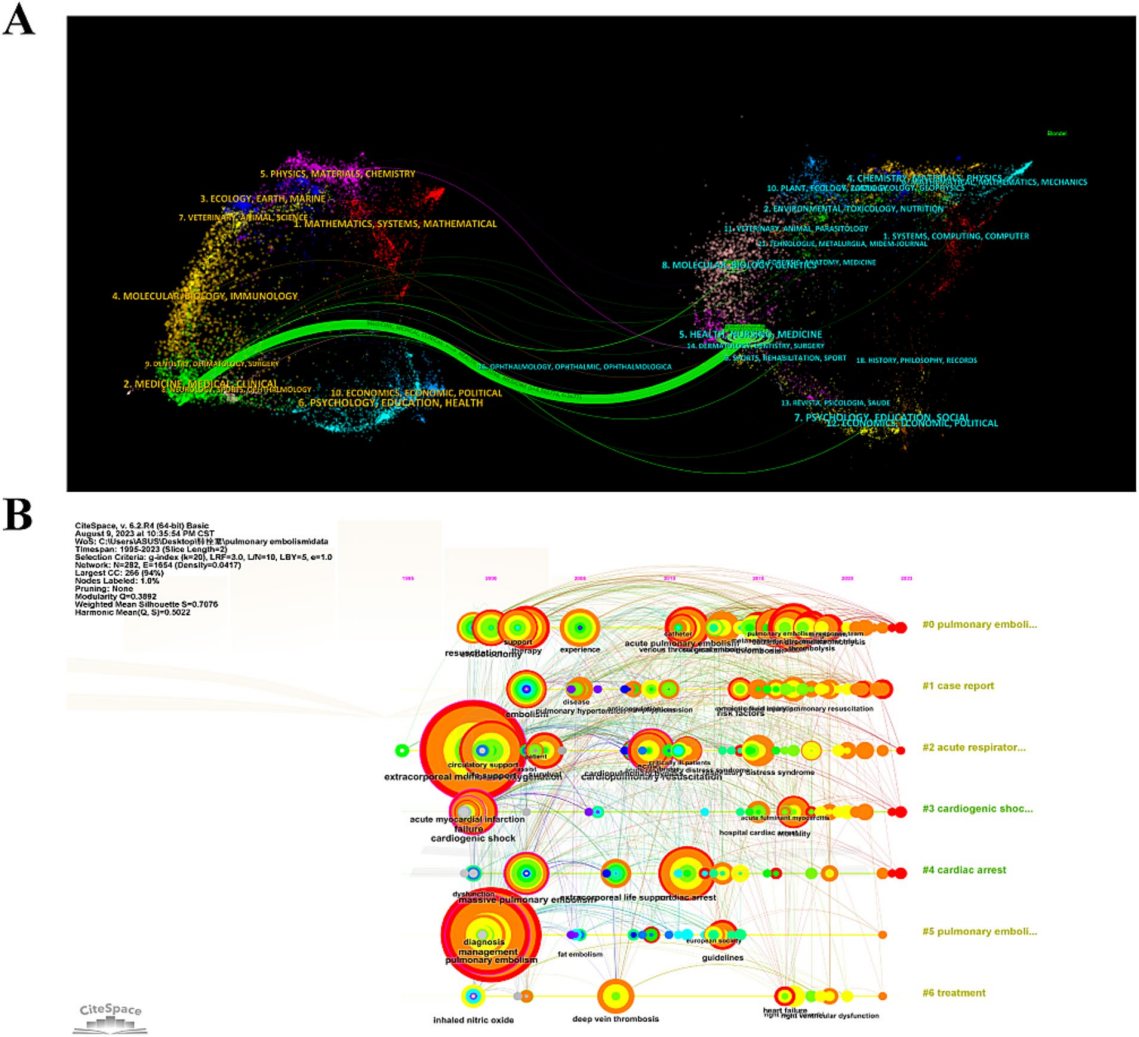
**(A)** Dual map of CiteSpace generated journals showing the thematic distribution of journals. Citing journals are located on the left-hand side of the map, while cited journals are located on the right-hand side of the map. The labels represent the disciplines covered by the journals. **(B)** The knowledge map of references related to research on ECMO-assisted PE.

The double mapping analysis illustrated the thematic distribution of academic journals (Figure 5A). The map depicted the cited journals on the left and the citing journals on the right, with colored lines denoting co-citation links. A singular green main line was observed, indicating a predominant trend where research published in health care/medical/nursing journals is also predominantly cited by studies in health care/nursing/medical domains.

We developed a co-citation reference timeline, as depicted in Figure 5B. This timeline view employs a visual approach that integrates clustering with temporal segmentation. The arrangement of cluster labels, based on their chronological emergence post-clustering, reflects the evolution of research themes and their interrelations within the field. In this visualization, nodes aligned on a horizontal plane signify different years; nodes to the left indicate older references, while those to the right denote more recent ones. Horizontal lines on the same level encapsulate the entire collection of references within a given cluster. The designation of the cluster is positioned at the extreme right of the temporal axis. At the forefront of the timeline is cluster “#0 pulmonary embolism,” succeeded by “#1 case report,” “#2 acute respiratory distress syndrome,” “#3 cardiogenic shock,” “#4 cardiac arrest,” and “#5 treatment.”

### Authors and co-cited authors

**Figure 6:**
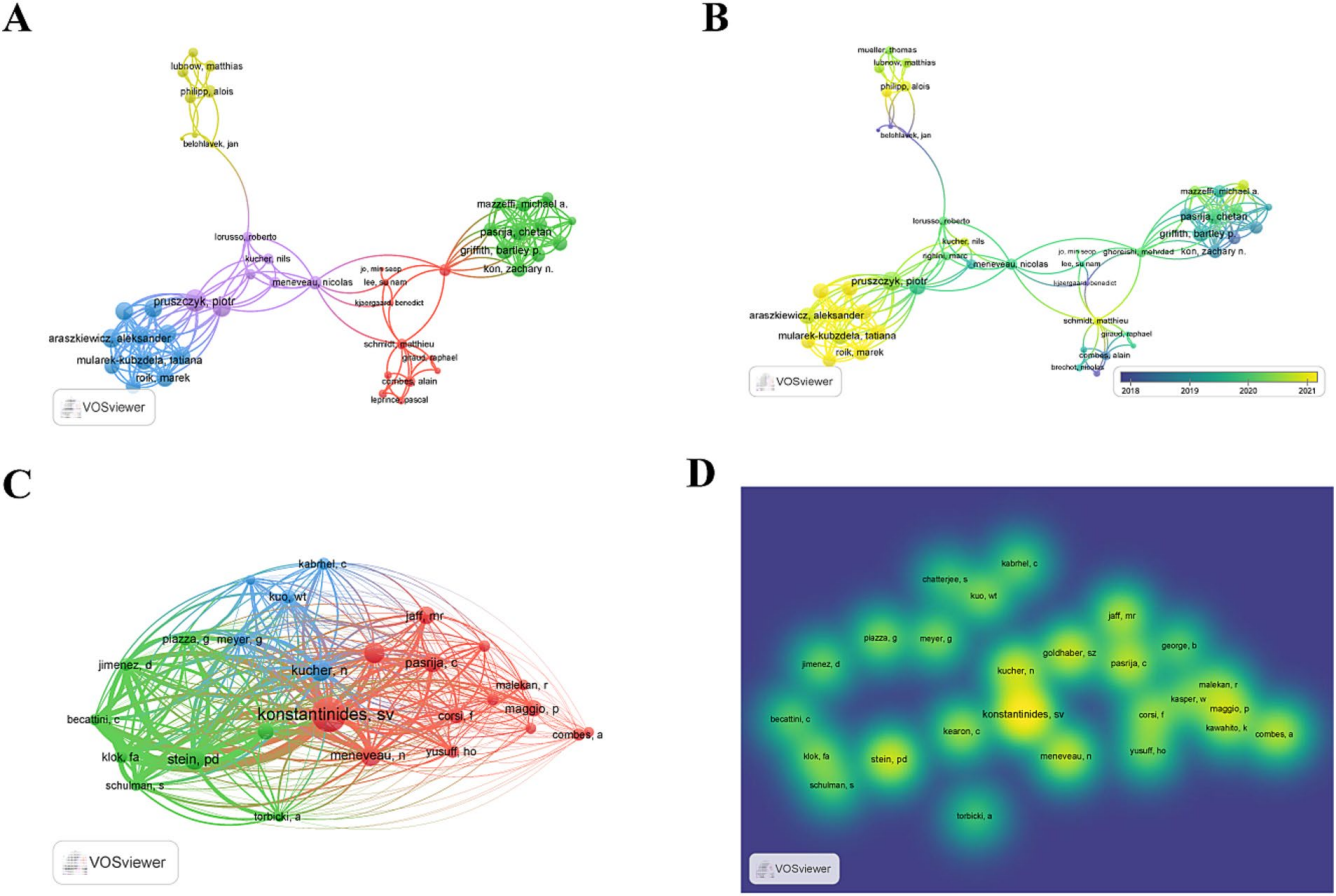
**(A,B)** Visual map of authors generated by VOSviewer (authors with ≥3 total publications); **(C,D)** Visual map of co-cited authors generated by VOSviewer (co-citation threshold ≥30 times).

A total of 2,288 authors have contributed to the scholarly discourse on ECMO-assisted research for PE. Among these, 76 authors have published three or more articles each. Notably, three authors have each published six articles (3.94%), and five authors have published five articles each (6.58%). An author network map was constructed based on a minimum publication threshold of three papers (Figures 6A,B). Leading the publication count was Chetan Pasrija from the United States, followed by Combes Alain from France and Roberto Lorusso from Netherlands (Table 5). In Figure 6B, the clustering of Aleksander Araszkiewicz, Tatiana Mularek-Kubdzaniewska, and Marek Roik in yellow indicates their recent entry into the field and their active publication record.

Among the 7,094 co-cited authors, Konstantinidis SV had the highest citation count (*n* = 159), followed by Paul D Stein (*n* = 89), Nils Kucher (*n* = 86), Samuel Z Goldhaber (*n* = 84), Nicolas Meneveau (*n* = 80), and Chetan Pasrija Combes (*n* = 75) (Table 5). A density map was generated for authors with co-citations exceeding 30 times (*T* ≥ 30) (Figures 6C,D). The map highlighted, in the most intense color, Konstantinidis SV as the most cited author.

**Table 5:** Top 10 authors and co-cited authors.

Rank	Author	Documents	TC	AAC	TLS	Co-cited author	TC	TLS
1	Chetan Pasrija	6	168	28.0	31	Stavros V Konstantinides	159	1,775
2	Alain Combes	6	224	37.3	13	Paul D Stein	89	1,671
3	Roberto Lorusso	6	2,313	385.5	13	Nils Kucher	86	1,103
4	Piotr Pruszczyk	5	2,295	459.0	42	Samuel Z. Goldhaber	84	754
5	Bartley P. Griffith	5	168	33.6	31	Nicolas Meneveau	80	944
6	Nicolas Meneveau	5	2,383	476.6	18	Chetan Pasrija	75	729
7	Alois Philipp	5	69	13.8	17	Michael R Jaff	68	588
8	Jay Giri	5	205	41.0	5	Clive Kearon	63	867
9	Aleksander Araszkiewicz	4	53	13.3	33	Paul Maggio	60	351
10	Tatiana Mularek-Kubzdela	4	53	13.3	33	Fillipo Corsi	56	33

### Top keywords

**Figure 7:**
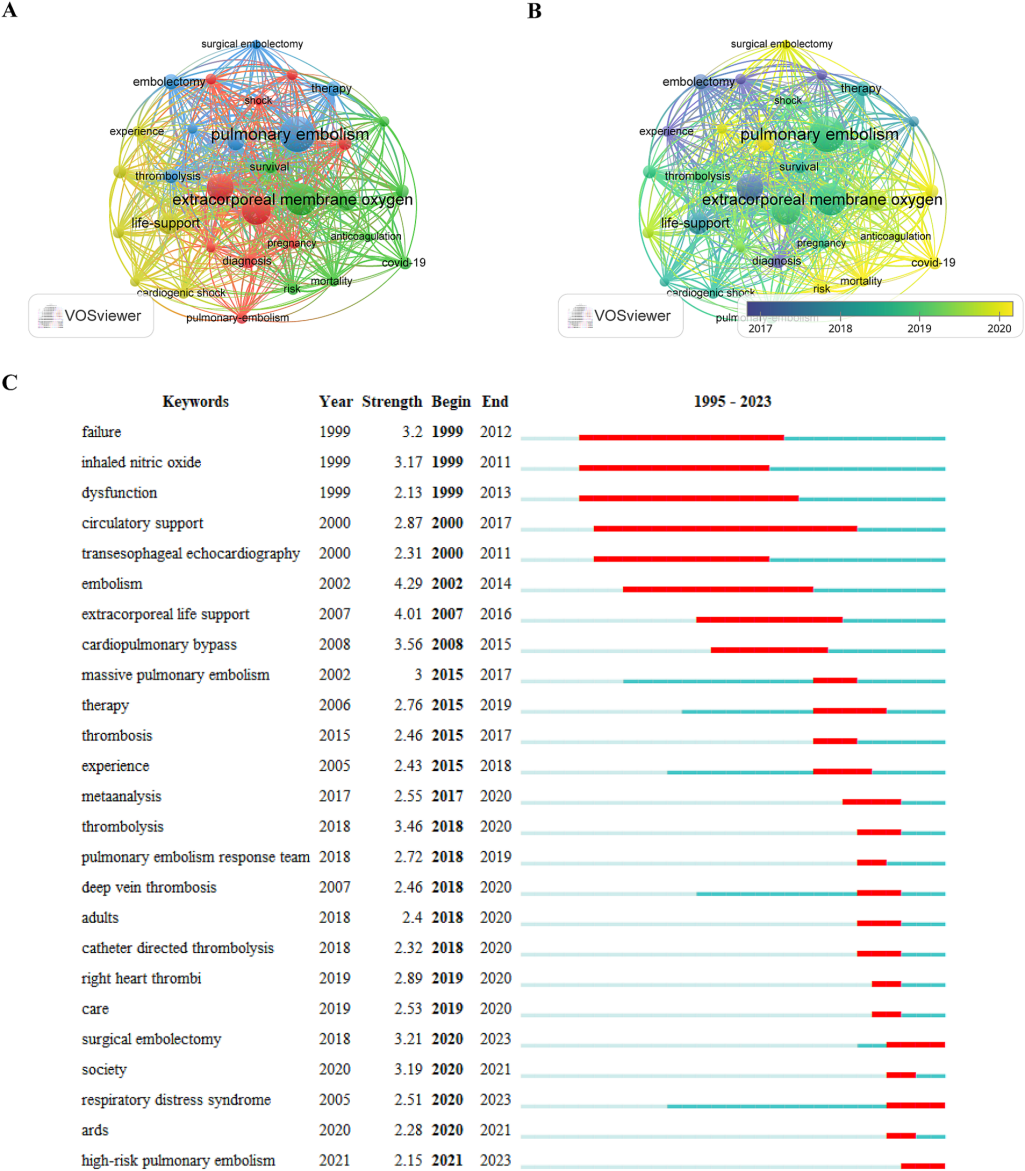
**(A,B)** Visual map of keywords generated by VOSviewer (frequency of occurrence ≥20 times); **(C)** Top 25 key burst words generated by CiteSpace.

Keyword graph and cluster analyses were meticulously conducted using VOSviewer (Figures 7A,B). This analysis yielded a total of 1,196 keywords, among which 32 appeared more than 20 times each. A frequency-density plot, a tool for identifying high-frequency co-occurring terms, is a highly useful method for identifying the prevailing themes within a specific research field. As depicted in Figure 7C and detailed in Table 6, “ECMO” emerged as the most prominent keyword, followed by “PE,” “management,” “life support,” “successful,” “embolization,” “thrombolysis,” “diagnosis,” “survival,” “blood clotting,” “acute heart failure,” “venous thromboembolism,” “COVID-19,” and “heart failure.” These terms collectively highlight the focal points and evolving trends in research on ECMO-assisted support for PE.

**Table 6:** Top 10 keywords.

Rank	Keyword	Occurrences	Total link strength
1	extracorporeal membrane oxygenation	195	635
2	pulmonary embolism	126	486
3	management	96	403
4	life-support	64	266
5	ECMO	62	184
6	outcomes	47	214
7	embolectomy	41	217
8	therapy	38	163
9	thrombolysis	34	192
10	diagnosis	34	139

### Cited references and top 20 highly cited references

**Figure 8:**
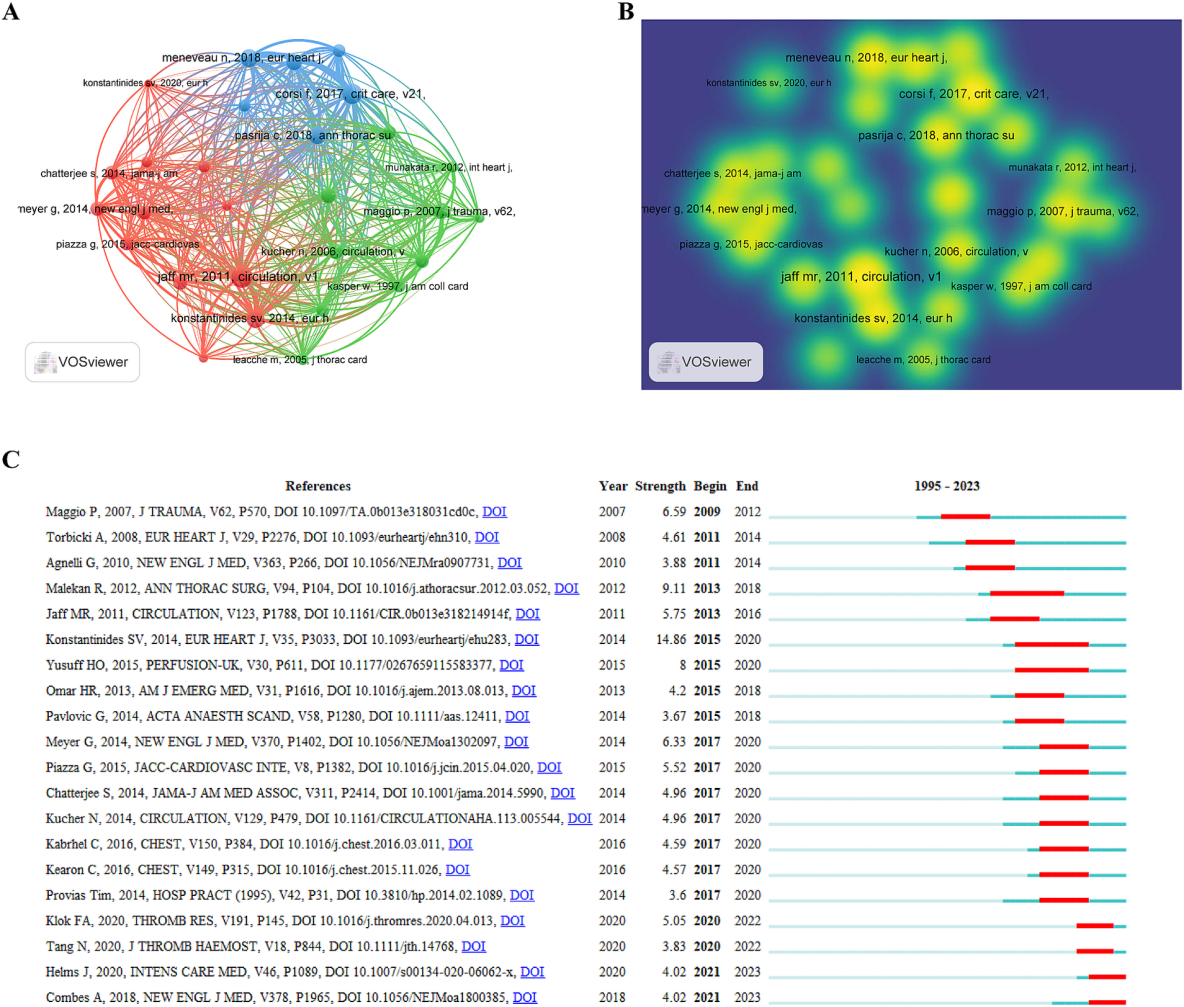
**(A,B)** Visual map of co-cited references generated by VOSviewer (co-cited references≥20); **(C)** Top 20 burst references generated by CiteSpace.

In our comprehensive analysis of cited references within the field of ECMO-assisted support for PE, we employed VOSviewer to identify the top 10 most-cited references from 1995 to 2023 (Figures 8A,B). The most cited article, authored by Jaff MR and published in *Circulation* in 2011, titled “Management of massive and sub-massive pulmonary embolism, iliofemoral deep vein thrombosis, and chronic thromboembolic pulmonary hypertension: a scientific statement from the American Heart Association,” garnered 68 citations. It was followed, with 60 citations, by “Extracorporeal life support for massive pulmonary embolism,” authored by Maggio P and published in the *Journal of Trauma and Acute Care Surgery* in 2007. The third most-cited work, by Stavros V Konstantinides in the *European Heart Journal* in 2014, was titled “2014 ESC Guidelines on the diagnosis and management of acute pulmonary embolism,” which received 57 citations.

CiteSpace was utilized to assess the highly cited references. The trend of increasing citations over time highlights the widespread recognition and enduring impact of these studies within the field (Figure 8C). Notably, among the top 20 highly cited studies, the 2014 publication of Konstantinides SV in the *European Heart Journal* (2015–2020, Strength 14.86) and the 2012 paper of Malekan R in the *Annals of Thoracic Surgery* (2013–2018, Strength 9.11) emerged as recent high-impact articles.

## Discussion

### Bibliometrics and visual analyses

Bibliometrics, a methodology for quantitative analysis for the review and investigation of literature within professional fields, traces its origins to the early 20th century (14). This analytical process provides detailed insights regarding published papers, including information related to authors, keywords, journals, countries, institutions, and references. Using computer technology, bibliometric analysis employs visual representations, such as graphs and charts, to render complex data more comprehensible and accessible (15).

Visual analysis, a key component of bibliometrics, elucidates the intricate relationships among various elements of scholarly work. It reveals common research themes shared by different authors, highlights the focal areas of research across institutions, detects emerging theories, and projects future research trajectories within a field (16). This approach not only simplifies the understanding of dense academic information but also provides a macroscopic view of the evolving trends and patterns in a specific domain.

### Trend regarding ECMO-assisted support for PE

The period spanning 1995 to 2010 saw the beginning of research on ECMO-assisted support for PE. This period was understandably characterized by the paucity of substantial evidence. However, as medical technology advanced over time, an increase in scholarly publications was noted, signaling the growing significance of this field of research. The advent of the COVID-19 pandemic in 2019 further accelerated this trend, as the incidence of PE cases rose globally, which in turn catalyzed more intensive research on ECMO-assisted support for PE. In 2022, the field reached its zenith with 68 publications. It is projected that research in this domain will continue to flourish in the coming years.

### Communication and cooperation between countries, institutions, and authors

The United States leads in publication volume globally, suggesting a correlation between research output and economic development. Developed countries, such as Germany, the United States, Italy, and the United Kingdom, exhibit higher total link strength (TLS), indicative of robust collaborative efforts in this research field. China, ranking third in article output, shows lower metrics in total citations, average citation rate, and TLS, pointing to a need for enhancing the quality of research and fostering international communication and cooperation. Among the publishing institutions, the University of Maryland leads the world in research in this field, which is inseparable from Chetan Pasrija—an ECMO expert at the university. He has published numerous studies on ECMO, all of which are authoritative. In the author network map, a uniform color denoting a single cluster signifies a robust correlation among authors within that cluster. For instance, the collaboration among Matthias Lubnow, Alois Philipp, Jan Belohlavek, all belonging to the same cluster, is notably positive. Moreover, intercluster collaborations are evident, as seen in the interactions among Nicolas Meneveau, Lee Su Nam, and Matthieu Schmidt. Each node in the network map represents an individual author, with the size of the node reflecting the author’s publication volume. The connecting lines symbolize co-occurrence relationships among authors; too many lines indicate frequent collaborations.

A pronounced co-occurrence relationship is observed between authors and their co-authors, with highly productive authors often engaging more frequently in collaborative efforts. An analysis of the data related to authors, citing authors, and cited literature showed that scholars, such as Konstantinides SV, Chetan Pasrija, Alain Combes, and Roberto Lorusso, are leading figures in this field. Their prominence and expertise suggest that these scholars and their teams could be valuable collaborators for future researchers in ECMO-assisted support for PE. It is imperative to emphasize the importance of fostering collaboration among authors, institutions, and nations. International cooperation is a potent driver for advancing knowledge and implementing practical changes in this field. Hence, there is a pressing need to actively enhance global collaborative efforts to spur innovation and development in ECMO-assisted support for PE research.

### Popular journals and development prospects

*Perfusion-UK* is at the forefront of international research and development on ECMO-assisted support for PE. It focuses on research in the field of peripheral vascular disease, providing the latest information on all aspects of perfusion, oxygenation, and biocompatibility and their application in modern cardiac surgery. *Circulation* is one of the world’s top journals and the most co-cited journal. It generally publishes scientifically excellent original research manuscripts, review articles, and other content related to clinical and laboratory research on cardiovascular disease, including observational studies, clinical trials, epidemiology, health services, and outcome studies, as well as advances in basic and translational research. Our study findings reveal that a significant proportion of articles on ECMO-assisted support for PE are published and cited in esteemed journals, reflecting the increasing global scientific interest in this domain. This trend also underscores the keen focus of these journals on research related to ECMO-assisted support for PE. Such insights are invaluable for future researchers in selecting appropriate journals for publishing their work in this field. Notably, countries with leading journals and prolific publications, such as the United States, are at the forefront of this research field. To clarify the positioning of ECMO-supported PE research in comparison to the interrelationships between major research disciplines and journals, a dual mapping overlay of journals was constructed. The dual citation curve fully depicts the intricacies of citation dynamics. We emphasized robust and seamless citation connections using the Z-score. Notably, the research on ECMO-assisted support for PE mainly tends to focus on the development of the clinical discipline. We suggest that future research in this field should be directed toward strengthening basic research and integrating multidisciplinary management approaches.

### Most popular articles on ECMO-assisted support for PE

The most cited article in the field of ECMO-assisted support for PE is “Extracorporeal life support for massive pulmonary embolism” by Maggio P, published in 2007 (17). This seminal study conducted a comprehensive review of case data from 21 patients who underwent ECMO treatment for massive PE between 1992 and 2005. Within this cohort, 19 patients were treated with VA-ECMO and 2 with VV-ECMO. The average duration of support for survivors was 5.4 days, ranging from 5 h to 12.5 days. Notably, the overall survival rate was 62% (13 out of 21 patients). This research highlighted the potential of ECMO as a life-sustaining intervention for critically ill patients with severe PE, especially those who are unable to tolerate other forms of intervention or treatment. At the time of its publication, this study represented the largest reported cohort of ECMO treatment for PE. The co-citation analysis of such highly cited literature offers invaluable insights, enabling a deeper understanding of the evolving knowledge structures in ECMO-assisted treatment for PE.

### Research trends and progress of ECMO-assisted support for PE

Over the past two decades, the application of ECMO as a supportive therapy in the treatment of PE has undergone significant advancements and evolution. By analyzing the progression of research hotspots, it is evident that the role of ECMO in the management of PE has transitioned from initial exploratory studies to more refined clinical applications, ultimately contributing to the establishment and optimization of standardized treatment protocols. Based on the distribution of research keywords, the studies on ECMO-supported PE management can be categorized into three major phases, with the keywords from each phase reflecting distinct focal points and technological advancements.

### Phase I (before 2007): exploring the feasibility of ECMO in PE management

From 1999 to 2007, research primarily focused on investigating the feasibility and early technical applications of ECMO for supporting patients with severe PE. Keywords such as “failure,” “inhaled nitric oxide,” “dysfunction,” “circulatory support,” and “transesophageal echocardiography” reflect the preliminary exploration of ECMO’s role in addressing cardiopulmonary failure during this period. PE, as an acute and potentially fatal vascular disease, is often accompanied by acute right ventricular dysfunction and severe circulatory collapse, rendering conventional treatments such as anticoagulation, thrombolysis, and surgical embolectomy insufficient in certain critical cases. During this time, ECMO emerged as a temporary extracorporeal circulatory support modality, garnering increasing attention from the critical care field for its potential to address the limitations of traditional therapeutic approaches.

In 1995, M. J. Davies reported a case of ECMO application in an awake patient with acute massive PE, achieving favorable outcomes while avoiding the risks associated with general anesthesia and open pulmonary embolectomy. This case expanded the scope of ECMO’s role in the circulatory management of acute massive PE (18). In 2001, a 58-year-old woman presenting with acute cardiopulmonary failure due to massive PE underwent pulmonary angiography and open surgical embolectomy under ECMO support, demonstrating strong evidence for ECMO’s feasibility in such critical situations (19). In 2006, Meneveau et al. published a prospective single-center study on patients with PE undergoing thrombolysis, highlighting that surgical embolectomy combined with ECMO support could serve as an effective rescue strategy following thrombolysis failure in acute massive PE (20). Similarly, in 1999, Von Segesser LK analyzed ECMO’s use in severe PE cases, emphasizing its potential as a lifesaving intervention in specific scenarios, such as neonatal and pediatric cardiopulmonary support systems. However, sepsis was considered a contraindication for ECMO at that time (21). A new case reported in an emergency medicine journal during this phase underscored the benefits of early ECMO initiation for critically ill PE patients, suggesting improved prognoses with prompt circulatory support (22). Research during this period primarily focused on exploring ECMO’s ability to provide circulatory support for patients with acute PE-induced right ventricular failure, especially in cases where significant right ventricular dysfunction rendered conventional treatments ineffective. Keywords such as “failure” and “dysfunction” indicate the emphasis on addressing respiratory and circulatory system failure in severe PE cases during this early stage.

Acute PE can lead to right ventricular (RV) failure and pulmonary hypertension, making hemodynamic stabilization a critical focus of management. Inhaled nitric oxide (iNO), a pulmonary vasodilator, has been extensively studied as an adjunct to ECMO, aiming to reduce pulmonary vascular resistance, alleviate pulmonary hypertension, decrease RV workload, and improve oxygen saturation. Research on the combined use of iNO and ECMO has primarily concentrated on conditions such as persistent pulmonary hypertension of the newborn (PPHN) and acute respiratory distress syndrome (ARDS). In 1994, Finer, N., demonstrated the efficacy of combining iNO with high-frequency ventilation in treating severe PPHN, noting that some patients required ECMO support and achieved favorable outcomes (23). Konduri, G., in a randomized trial, compared early versus standard iNO therapy for term and near-term neonates with hypoxemic respiratory failure, revealing that early iNO use reduced the need for ECMO (24). Similarly, Davidson, D., in a 1998 multicenter randomized controlled study, evaluated the effects of varying doses of iNO in term neonates with PPHN, with ECMO support utilized in certain cases, thereby providing critical evidence for the combined application of iNO and ECMO in managing severe respiratory failure (25).

Additionally, transesophageal echocardiography (TEE) emerged as a pivotal diagnostic and monitoring tool during this period. TEE enabled real-time assessment of RV function and hemodynamic changes under ECMO support, offering invaluable guidance for clinical decision-making. In 1995, Marcus B. reported on the application of TEE in pediatric patients requiring ECMO post-cardiac surgery. The study highlighted TEE’s ability to provide accurate diagnostic information when transthoracic echocardiography was limited, allowing more effective evaluation and treatment guidance (26). TEE also proved instrumental in identifying complications during ECMO cannulation, such as mispositioning of venous cannulas through the atrial septum into the left atrium, and guiding their repositioning into the inferior vena cava (27). Moreover, TEE has been validated as a highly effective bedside tool for diagnosing suspected massive PE. It provides clear visualization of the pulmonary artery trunk, right pulmonary artery, and proximal left pulmonary artery. Skilled clinicians can use TEE-derived anatomical and functional information to assess ventricular function and guide timely thrombolytic interventions (28). Overall, research during this phase predominantly focused on elucidating the pathophysiological mechanisms underlying acute PE and exploring the feasibility of ECMO’s initial applications. These investigations laid the foundation for subsequent advances in ECMO-assisted PE management.

### Phase II (2008–2017): expansion of ECMO use and optimization of treatment strategies

Between 2008 and 2017, advancements in technology and the successful application of ECMO in critically ill patients shifted its role in PE from theoretical exploration to practical clinical implementation. During this phase, research focused on expanding the indications of ECMO and optimizing therapeutic strategies for different types of PE. Keywords such as “embolism,” “extracorporeal life support,” and “cardiopulmonary bypass” highlighted the growing trend of ECMO as an essential emergency intervention, its integration with traditional extracorporeal circulation techniques, and its significance in maintaining life support. Benjamin and Assouline provided insights into the application of VA-ECMO in the management of high-risk PE, evaluating its efficacy as a standalone therapy or a bridge to other treatments. They emphasized that despite the increasing use of VA-ECMO, the evidence base remained limited, necessitating prospective randomized trials to compare its outcomes with systemic thrombolysis (29). Rasha Al-Bawardy and colleagues summarized their experience with ECMO in patients experiencing massive PE complicated by cardiac arrest. They reported that ECMO, in combination with systemic thrombolysis, catheter-directed therapy, or surgical embolectomy, improved survival chances despite a 30-day mortality rate of 31% and a mortality rate of 54% (30). Chetan Pasrija and colleagues conducted a retrospective analysis of patients with massive pulmonary embolism treated with VA-ECMO. The study included 20 patients, of whom 40% received anticoagulation therapy alone, 5% underwent catheter-directed therapy, and 55% underwent surgical pulmonary embolism removal. The in-hospital and 90-day survival rates were 95% (31). Fillipo Cors et al. analyzed 17 patients with high-risk PE treated by ECMO at the center and showed that the 90-day survival rate of patients included in the center was 47%. Fifteen of these patients (88%) developed serious complications in the ICU. In conclusion, these multicenter studies suggest that VA-ECMO may be an important treatment option for high-risk PE patients (32).

In cases of massive PE, which are often associated with severe hypoxemia and circulatory collapse, ECMO gained recognition as one of the most effective emergency support measures. João Valente, Jorge, and colleagues documented the successful rescue of a 49-year-old woman with massive PE using a combination of VA-ECMO and pulmonary aspiration thrombectomy. This report underscored the potential of ECMO to enhance systemic organ perfusion and improve survival during resuscitation while highlighting the importance of multidisciplinary collaboration for complex interventions (33). Another case study emphasized the necessity of continuous monitoring and systematic evaluation when using systemic thrombolytics during V-V ECMO support for massive PE. The study also underscored the utility of bedside echocardiography in assessing cardiac function during ECMO (34). Furthermore, the Pulmonary Embolism Response Team (PERT) emerged as a critical component in the management of massive and submassive acute PE. PERT was instrumental in determining optimal treatment strategies based on individual clinical presentations and facilitated the rapid implementation of mechanical thrombectomy or suction embolectomy, significantly improving survival rates in PE patients (35). As clinical experience with ECMO expanded, its integration with thrombolytic therapies, such as systemic thrombolysis and catheter-directed thrombolysis, began to be reflected in treatment guidelines. This combined strategy effectively reduced thrombus burden while ECMO provided vital life support, granting patients critical recovery time.

Concurrently, retrospective analyses and meta-analyses became prominent during this phase, laying a more robust evidence foundation for future clinical practice. Keywords like “meta-analysis” signified the growing emphasis on data synthesis to inform treatment strategies. Elona Rrapo Kaso and colleagues conducted a meta-analysis comparing in-hospital mortality rates between patients with acute massive PE who received ECMO and those who did not. Their findings indicated no significant difference in in-hospital mortality between the two groups (odds ratio = 1.24 [95% CI, 0.63–2.44], *p* = 0.54) (36). For high-risk PE patients, the primary indication for ECMO was cardiac arrest, with an early all-cause mortality rate of 41.1%. Major bleeding was identified as the most common adverse event during hospitalization (37). The team led by Matteo Pozzi reviewed and assessed clinical outcomes of high-risk PE patients treated with ECMO. Despite substantial heterogeneity in both in-hospital and follow-up survival rates, the current body of literature remains insufficient to definitively establish ECMO’s role in managing high-risk PE. The authors highlighted the urgent need for prospective, multicenter, and large-scale studies to clarify ECMO’s efficacy and optimize its application in high-risk PE management (38).

During this phase, multidisciplinary team models, exemplified by the Pulmonary Embolism Response Team (PERT), emerged as pivotal to improving the management of PE. The collaboration of multiple specialties, including cardiothoracic surgery, critical care, anesthesiology, and interventional radiology, became essential for the successful implementation of ECMO. Under this framework, ECMO was gradually integrated into comprehensive PE treatment systems, leading to improved coordination of emergency resources, optimization of diagnostic and therapeutic workflows, and enhanced treatment efficiency and patient survival. Dudzinski, D. outlined the composition and functions of multidisciplinary PERTs, emphasizing their role in evaluating and implementing advanced therapies such as ECMO during decision-making processes (39). In 2017, Kabrhel, C. reported preliminary experiences with a multidisciplinary PERT, highlighting the team’s crucial role in the rapid assessment and treatment of high-risk PE patients, including the utilization of ECMO (40). Further, Rosovsky, R. P. analyzed changes in treatment strategies and patient outcomes before and after the establishment of a PERT. Their findings demonstrated that introducing PERT improved survival rates in high-risk PE patients and facilitated the application of advanced therapies, including ECMO (41). These studies underscored PERT’s critical role in the evaluation and management of high-risk PE cases, particularly in determining the use of advanced treatments like ECMO.

In addition, keywords such as “right heart thrombi” and “deep vein thrombosis” signified the application of ECMO in managing complex cases involving right heart thrombi and deep vein thrombosis with PE. Chen et al. reported a case of a patient with deep vein thrombosis in the right leg and a right atrial thrombus causing PE. They successfully treated the patient using an AngioJet device and VA-ECMO, demonstrating the feasibility and effectiveness of this combined approach in PE management (42). Similarly, E. Rodriguez-Ruiz and colleagues managed a 44-year-old male with severe refractory respiratory failure caused by bilateral pulmonary contusions due to chest trauma. During VV-ECMO cannulation via the femoral vein, a massive thrombus was observed in the right atrium extending through the tricuspid valve into the pulmonary artery. Despite cardiac arrest during the procedure, the patient was resuscitated with ECPR and ultimately recovered with no neurological deficits after 7 days of ECMO support (43). Between 2008 and 2017, ECMO transitioned from a theoretical concept to a clinical reality in PE management, with research expanding its indications, optimizing strategies, and highlighting its potential as a life-saving intervention despite limited evidence and significant complications.

### Phase III (2018–2023): multicenter studies and management of high-risk patients

After 2020, the application of ECMO-assisted support in PE entered a new developmental phase. The focus shifted toward optimizing ECMO use to enhance its efficacy and reduce complications. Keywords such as “ARDS,” “high-risk pulmonary embolism,” and “society” highlighted ECMO’s expanding role in managing complex cases. This phase marked the emergence of multicenter studies and the formation of internationally standardized and protocolized treatment approaches. A significant research focus during this period was the use of ECMO in patients with ARDS complicated by PE. ARDS frequently co-occurs with PE, particularly in high-risk patients. ECMO not only provides circulatory support but also improves severe hypoxemia through membrane oxygenation. The citation surge of the keyword “ARDS” reflects this trend, showcasing ECMO’s dual role in managing both respiratory and circulatory failure. Guillaume Lebreton et al. collected all laboratory-confirmed SARS-CoV-2 infections and severe ARDS adult patients requiring ECMO from 7 Greater Paris intensive care units. Pulmonary embolism occurred in 53 of 294 patients (18%). 138 patients (46%) survived 90 days after ECMO. The most common causes of death were multiple organ failure and septic shock (44). Another study published by Nicolas Meneveau retrospectively included 180 high-risk PE patients from 13 departments at 9 centers. Fifty-two patients received ECMO. The overall 30-day mortality was 48.3%. Among high-risk PE patients, ECMO group had severe clinical manifestations and poor prognosis. But ECMO shows promise as a complementary treatment to surgical embolectomy (45).

In 2019, Ayman Elbadawi et al. analyzed trends in ECMO use among high-risk PE patients using a national database of inpatient samples. They found that ECMO use increased during the study period and that ECMO use was associated with a reduction in mortality in patients with extensive PE (*p* < 0.001). The in-hospital mortality rate for ECMO patients was 61.6% (46). Nikhil K. Prasad retrospectively collected data on 83 patients with PE who required VA-ECMO support between December 2015 and December 2020. The results showed no statistically significant difference in the discharge survival rate between patients who received surgical thrombectomy (ST) and those who did not (88.9% vs. 84.6%; *p* = 0.94). However, the incidence of major bleeding events was higher in patients who underwent ST (61.1% vs. 26.2%; *p* = 0.01) (47). After 2020, ECMO-assisted support in PE evolved with a focus on optimizing efficacy and reducing complications, particularly in ARDS-complicated cases, as highlighted by multicenter studies showing ECMO’s increasing role, survival outcomes, and associated risks.

Yang et al. successfully treated an adult patient with fulminant psittacosis-induced severe ARDS who experienced near-fatal PE and cardiac arrest during VV-ECMO support. This case demonstrated VV-ECMO’s efficacy as an emergency life-saving intervention for severe ARDS caused by fulminant psittacosis. The study also emphasized the importance of routine monitoring during ECMO to promptly detect and manage thrombosis, thereby preventing catastrophic events like PE and cardiac arrest (48). During the COVID-19 pandemic, some patients presented with a combination of respiratory and vascular complications, including ARDS and PE. Khawaja M. Talha and colleagues described a male patient with ARDS and bilateral PE, whose severe hypoxemia persisted despite maximum flow on VV-ECMO. A second venous cannula was introduced to form a unique V-V-V ECMO circuit, successfully resolving refractory hypoxemia. This case highlighted the critical importance of simultaneously addressing thrombotic risks and shock in COVID-19 patients to improve outcomes and mitigate ECMO-related complications such as right ventricular failure (49). This phase underscores the progression of ECMO from circulatory support to comprehensive management of respiratory and thrombotic complications, particularly in high-risk and complex clinical scenarios. The rise of multicenter research and standardized protocols has further solidified ECMO’s role as an indispensable tool in managing high-risk PE.

With the maturation of ECMO technology, a growing consensus has emerged regarding its application in managing high-risk PE. Keywords such as “high-risk pulmonary embolism” and “surgical embolectomy” reflect this phase’s focus on providing more personalized treatment options for high-risk patients, especially in cases where conventional therapies fail or are contraindicated. Surgical embolectomy combined with ECMO support has become an increasingly favored clinical approach. Multiple studies have analyzed the adjunctive role of ECMO in surgical embolectomy, exploring how it can support high-risk patients unable to tolerate traditional thrombolytic therapy. Corsi, F. et al. investigated the use of ECMO in patients with life-threatening massive PE, emphasizing its role as a life-saving rescue therapy when thrombolysis fails or surgical embolectomy is not feasible (32). Meyer, M. conducted a literature review in 2018, highlighting ECMO’s critical role in supporting surgical embolectomy, particularly for hemodynamically unstable patients with massive PE (30).

The keyword “society” further signifies the global effort by professional organizations and experts to develop consensus and guidelines for ECMO in PE treatment. For instance, the 2019 European Society of Cardiology (ESC) Guidelines on the Diagnosis and Management of Acute PE detailed diagnostic and therapeutic strategies, recognizing ECMO as a circulatory support option for high-risk patients, particularly when conventional therapies are ineffective or contraindicated (50). Similarly, the 2020 American Heart Association (AHA) Guidelines for Cardiopulmonary Resuscitation and Cardiovascular Care emphasized ECMO’s role in extracorporeal cardiopulmonary resuscitation (ECPR), applicable in cardiac arrest cases caused by PE (51). In 2021, the Extracorporeal Life Support Organization (ELSO) Guidelines for Adult ECMO provided recommendations for ECMO use in various clinical scenarios, specifically identifying it as an essential support modality in cardiogenic shock caused by massive PE (52). Further advancing the field, the 2022 Chinese Expert Consensus on Adult ECMO-Assisted Circulatory Support tailored these guidelines to domestic clinical practice, detailing indications, contraindications, and procedural workflows for ECMO in acute PE management while emphasizing the importance of multidisciplinary collaboration. These consensus guidelines have not only promoted the broader application of ECMO but also laid the groundwork for future research. Efforts are now directed toward standardizing treatment protocols, minimizing complications, and improving long-term patient outcomes. These developments highlight ECMO’s increasingly pivotal role in the management of high-risk pulmonary embolism.

### Limitations and future perspectives

Although this study contributes to the advancement of ECMO-assisted support in the management of PE, several limitations must be acknowledged. First, some high-quality articles on ECMO-supported PE management may not have been fully captured in the Web of Science database. Literature from other databases, such as PubMed and Embase, was not guaranteed to be included in our search strategy, potentially introducing selection bias. Additionally, our inclusion criteria limited the review to studies published in English, excluding potentially relevant articles in other languages. Furthermore, this study primarily included literature published between 1995 and 2023, and the most recent research reports published after the cut-off date may not have been included. This study relies solely on the Web of Science Core Collection database and is limited to English-language literature, which may lead to the omission of non-English studies (e.g., Chinese, Japanese, or German literature). These studies may contain important regional or innovative research findings, potentially introducing a certain degree of bias in our results. Future research should incorporate multilingual literature and multiple databases to enhance the comprehensiveness and representativeness of the findings.

## Conclusion

In summary, the development of ECMO-assisted support in PE treatment has followed a comprehensive trajectory, evolving from foundational research to clinical application and the establishment of standardized protocols. Early studies provided theoretical groundwork, while accumulated clinical experience has offered empirical support for its broader use. Future research should focus on optimizing ECMO application protocols, reducing complication rates, improving long-term patient outcomes, and exploring synergistic combinations with emerging therapeutic modalities. These efforts aim to enhance the efficacy and safety of ECMO in the treatment of pulmonary embolism.

## Data Availability

The raw data supporting the conclusions of this article will be made available by the authors, without undue reservation.
